# Tree Shrew as a New Animal Model for the Study of Dengue Virus

**DOI:** 10.3389/fimmu.2021.621164

**Published:** 2021-03-25

**Authors:** Liming Jiang, Caixia Lu, Qiangming Sun

**Affiliations:** ^1^ Institute of Medical Biology, Chinese Academy of Medical Sciences, and Peking Union Medical College, Kunming, China; ^2^ Yunnan Key Laboratory of Vaccine Research & Development on Severe Infectious Diseases, Kunming, China; ^3^ State Key Laboratory for Managing Biotic and Chemical Threats to the Quality and Safety of Agro-products, Ningbo University, Ningbo, China

**Keywords:** dengue virus, tree shrew, animal model, dengue fever, viremia

## Abstract

Dengue virus is a significant public health threat worldwide; however, the pathogenesis of dengue disease remains poorly understood due to lack of appropriate small animal models. Tree shrews are an emerging experimental animal model for the study of human diseases due to their resemblance of genetic characteristics to primate animals. Herein we report that dengue infection in tree shrews elicits resemble clinical symptoms as in humans. Dengue fever (△2°C> normal body temperature) developed in ~22% healthy Chinese tree shrews from 2 through 33 days after infection with a low dose (1 ∗ 10^4^ PFU/animal) of dengue virus serotype 2 or 3 intravenously or subcutaneously. The dengue genomic RNA and neutralizing antibodies were detected in ~78% of animals at days 7 and 15 post infection respectively. The serum levels of liver enzymes including aspartate transaminase, alanine aminotransferase and alkaline phosphatase were elevated with peaks at day 7 after infection. Modest thrombocytopenia and a slight decrease in the white blood cell count were observed. Intriguingly, although viral RNA was barely detectable in the liver by 48 days after infection, it was still evident in the brain. The intra-brain bleeding lesions in the intravenous infection group were more severe than those in the subcutaneous infection group. Our data demonstrate that primary dengue virus infection in tree shrews causes resemble clinical disease as in humans and thus tree shrews may be a suitable model for the study of dengue disease pathogenesis.

## Introduction

Dengue infection is one of the major public burdens in the tropical and subtropical areas worldwide, particularly in south Asia, Africa, central and south America. Approximately 3.6 billion people in these areas are at risk for epidemic transmission. Approximately 400 million people in more than 100 countries are infected with dengue each year (1). This significant public health threat is no longer confined to the traditional districts, and autochthonous dengue transmission has now been recorded in several European countries (2). In 2014, Japan reported its first outbreak of dengue in 70 years (3).

Despite the public health significance of dengue disease, there are still no highly effective vaccines and antiviral drugs. A major obstacle to the development of vaccines and therapeutics is the lack of validated animal models that recapitulate human infection. Primates are the only natural vertebrate hosts for DENVs. DENV infection in non-human primates (NHPs) results in acute viremia and a strong neutralizing antibody response; however, no overt clinical symptoms develop, such as hemorrhage, fever and shock (4, 5). Even many recent efforts have been made to improve the NHP models (6), the majority of the studies have not shown promising clinical disease (7). Moreover, the ethical concerns and high cost significantly make it unfeasible to apply NHPs to widespread DENV research.

Chinese tree shrews are mainly distributed across southwestern China (including the Yunnan, Sichuan, Hainan, Guizhou, and Guangxi provinces) and have been classified as *Tupaia belangeri*. Phylogenetic analysis of the Chinese tree shrew supports its close relationship to primates, and this animal is more resemble to humans than murine species, such as mice, marmots, and rats, in terms of its genomics (8). Moreover, Chinese tree shrews have other advantages, such as low cost, availability and easy maintenance. Thus, the Chinese tree shrew may be a promising animal model for human diseases. The tree shrew model is more frequently used in biomedical research as a replacement for primates in many human disease research, such as HCV, HBV, HEV, HSV, depressive disorder, thrombus and glioblastoma (9–11). The tree shrew T cells are genetically closely related to those of humans. A member of the flaviviruses, Zika virus, has been recently shown to induce clinical symptoms in tree shrews (12, 13), suggesting that tree shrews may be a good model for dengue virus research.

In this study, we explore the feasibility of applying tree shrews to dengue research. We observe clinical symptoms including fever, viral infection, high antibody titers and pathology in the central nervous system after primary infection. These symptoms largely recapitulate dengue infection in humans.

## Materials and Methods

### Phylogenetic and Identity Analysis of STING Gene Among Tree Shrew, Homo and Rattus Species

The reference STING (stimulator of interferon genes), TLR3 (Toll-like receptors) and MDA5 (melanoma differentiation-associated protein) genes sequences that were used to construct the distinct phylogenetic branches and identity analysis were collected from the GenBank sequence database of National Center for Biotechnology Information under the following accession numbers: *Homo sapiens* (MF622062.1, DQ360815.1 and AF095844.1), *Rattus norvegicus* (NM_001109122.1, NM_001109199.1 and XM_008771267.2), *T. belangeri* (KU998263.1, XM_006160265.3 and XM_006160465.2), *Macaca mulatta* (MF622060.1, NM_001036685.1 and DQ875603.1), *Sus scrofa* (MF358967.1, KT735340.1 and FJ455509.1) and *Mus musculus* (KR154221.1, NM_001357317.1 and NM_001164477.1). STING gene of tree shrew in this study was amplified from spleen tissue, followed by sequencing.

The phylogenetic analyses of STING and MDA5 gene were performed using the MEGA version 7 software and the phylogenetic trees were assembled using the Neighbor-Joining method of the Maximum Composite Likelihood model (14). The branching pattern was statistically evaluated by bootstrap analysis of 1,000 replicates. The identity analyses among tree shrew, Homo and Rattus species were performed using the Bioedit software.

### Culture of Primary Tupaia Bone Marrow Mesenchymal Stem Cells and DENV Infection

DENV-2 (KR870423.1) and DENV-3 (KY911981.1) were isolated from Guangzhou City, Guangdong Province, China and propagated in *Aedes albopictus* mosquito (C6/36, at 16th generations). The cell line was maintained in RPMI-1640 medium (1640, BI) supplemented with 10% fetal bovine serum (FBS, SJQ), 100 μg/ml streptomycin and 100 U/ml penicillin at 28°C in 5% CO_2_. The bone marrow mesenchymal stem cells (BM-MSCs) isolated and cultured from femur and tibia of tree shrew were as previously described (15). MSC plated in 24-well plates at 5 × 10^5^ cells per ml were cultivated in DMEM medium and incubated 24 h at 37°C in 5% CO_2_ in a humidified incubator. Twenty-four hours later, the DENV-3 was added in BM-MSCs at a multiplicity of infection (MOI) of 0.1. Cells were infected in triplicate and collected at 0, 12, 24, 36, 48, 60, 72, 84 and 96 h post infection. Vero cells were used as positive controls. The culture supernatants were collected for DENV RNA load determination using qRT-PCR. qRT-PCR was performed using SYBR Premix Ex Taq II (TaKaRa Bio, China). The standard curve of qRT-PCR was y = −0.3062 x + 12.054 (y = log10, x = Ct).

### Tree Shrews

Adult wild male (N = 21) and female (N = 24) Chinese tree shrews (N = 45, weighing 110−200 g and normal body temperature 36–37.5°C were obtained from Lufeng, Yunnan, China at the primate of medicine research center, Institute of Medical Biology, Chinese Academy of Medical Sciences. All animals were provided free access to food and water. All animal care and experimental protocols were approved by the Animal Care and Use Committee of Institute of Medical Biology, Chinese Academy of Medical Sciences, China.

### Injection

The tree shrews were anesthetized with ketamine. Then, 150 µl of DENV (DENV-3: 1 × 10^4^ PFU/ml, DENV-2: 1 × 10^4^ PFU/ml, diluent with RPMI-1640 medium (1640, BI)) was injected through intravenous injection or multisite subcutaneous injection (Abdomen, random of five sites, 30 µl/site). Complete the operation, the tree shrew was put into the ventilation area to restore, and all tree shrews were provided good care and recovered well (**Table 1**).

**Table 1:** Specifications of tree shrews.

Tree shrew	Sex	Age, Months	Weight, g	BT	Infection
TS1	M	36	148.35	36.5	Mock
TS2	M	48	139.75	37.1	Mock
TS3	F	15	139.7	37.2	Mock
TS4	F	44	147.1	36.4	Mock
TS5	M	47	151.05	36.4	Mock
TS6	M	36	132.55	36.5	Mock
TS7	M	48	127	36.6	Mock
TS8	F	24	129.05	36	Mock
TS9	F	36	139.6	37.2	Mock
TS10	M	7	136.5	36.6	DV-2-II-1
TS11	F	23	128.25	37.3	DV-2-II-2
TS12	M	24	156.15	37.1	DV-2-II-3
TS13	M	7	162.1	36.4	DV-2-II-4
TS14	M	19	148.95	37.5	DV-2-II-5
TS15	M	19	163.6	36.7	DV-2-II-6
TS16	F	23	113.6	37.1	DV-2-II-7
TS17	F	24	126.5	36.2	DV-2-II-8
TS18	M	26	132.9	36.5	DV-2-II-9
TS19	F	23	120	37.37	DV-2-SMI-1
TS20	F	23	126.1	36.8	DV-2-SMI-2
TS21	M	27	162.45	36.5	DV-2-SMI-3
TS22	F	26	138.85	37.1	DV-2-SMI-4
TS23	F	28	128.6	37.4	DV-2-SMI-5
TS24	F	24	126.75	36.5	DV-2-SMI-6
TS25	F	27	117.45	36.3	DV-2-SMI-7
TS26	F	23	138.35	36.6	DV-2-SMI-8
TS27	F	26	123.8	36.5	DV-2-SMI-9
TS28	F	48	154.45	36.5	DV-3-II-1
TS29	F	44	138.9	36.3	DV-3-II-2
TS30	M	7	145.5	36.7	DV-3-II-3
TS31	M	36	155.65	36.5	DV-3-II-4
TS32	F	7	126.8	36.8	DV-3-II-5
TS33	M	55	135.3	36.3	DV-3-II-6
TS34	M	6	139	36.4	DV-3-II-7
TS35	F	48	135.85	36.1	DV-3-II-8
TS36	M	6	127.85	36.7	DV-3-II-9
TS37	M	30	166.4	36.2	DV-3-SMI-1
TS38	F	16	147.95	36.5	DV-3-SMI-2
TS39	F	30	144.9	36	DV-3-SMI-3
TS40	F	18	129.3	37.1	DV-3-SMI-4
TS41	M	56	176	36.4	DV-3-SMI-5
TS42	F	24	134.05	36.5	DV-3-SMI-6
TS43	M	49	151.5	36.4	DV-3-SMI-7
TS44	F	17	140.3	36.3	DV-3-SMI-8
TS45	M	24	135.7	36.7	DV-3-SMI-9

All tree shrews used in this study were Chinese tree shrews.

The animals were sacrificed on day 48 after infection.

Mock group were infected with media used for C6/36 cell culture.

BT, body temperature; SMI, subcutaneous multipoint injection; II, intravenous injection.

### Weight and Temperature

The weight and temperature of tree shrews were measured on the 2nd, 7th, 15th, 21st, 28th, 35th, 41st and 48th days through electronic scales in a muffler and infrared thermometer on anus with three repetitions.

### Blood and Tissue Sample Collection

Blood samples were collected on the 2nd, 7th, 15th, 21st, 28th, 35th, 41st and 48th days after DENV-2/-3 infection (tail vein blood, 0.4 ml each time). The DENV detection in liver and brain biopsies were performed on the 48th day under anesthesia with ketamine hydrochloride and 1% pentobarbital. Serum samples were separated from the collected blood through centrifugation at 3,500 rpm at 4°C. Viral RNA was extracted from 150 μl of tree shrew serum or liver and brain tissue extracts using the QIAamp viral RNA mini kit (Qiagen, Hilden, Germany) and eluted in 50 μl of nuclease-free water. The extracted RNA was used for RT-PCR amplification to calculate viral RNA copy number titration (16). The residue of each liver and brain biopsy sample was frozen immediately by immersion in liquid nitrogen and then stored at −80°C before use.

### Neutralizing Titer Examination of Tree Shrew Serum

The standard method of antibody titer determination was used. Briefly, twofold serial dilutions of DENV2/3 immune serum (collected on the 2nd, 7th and 15th days) were prepared with RPMI-1640 medium (1640, BI). A mixture of 0.05 ml of each dilution degree and DENV-3/2 (200 TCID50) was maintained at 37°C for 2 h and then added to every well of a 96-well plate, with saturability for 95% of the C6/36 cell line. The cells were cultured for 7 days at 28°C and 5% CO_2_, and examinations were conducted at 5 and 7 days with a microscope. The endpoint is to quantify viral titer by determining the concentration at which 50% of the infected cells display cytopathic effect.

### Biochemical Markers and Routine Blood Examination

The serum of tree shrews was collected on the 2nd, 7th, 15th, 21st, 28th, 35th, 41st, and 48th days after intravenous and multisite subcutaneous injection. The biochemical markers and routine blood examination were conducted by the Ministry of Experimental Animals, Institute of Medical Biology, Chinese Academy of Medical Sciences (Automatic biochemical analyzer (BS-200) and Automatic blood analyzer (XT-2000i)).

### Histological Analysis in the Brain and Liver of Tree Shrews After Infection With DENV-3

The tree shrews brain tissues were collected from anesthetized animals at 48th day after intravenous and multisite subcutaneous injection of DENV virus, and then fixed with 10% neutral buffered formalin, embedded in paraffin, sectioned at 4 μm and stained with hematoxylin and eosin (H&E) after dehydration (17). The pathological sections were examined and photographed under a microscope (Nikon, ECLIPSE Ni).

### Ethics Statement

All animal care and experimental protocols were approved by the Animal Care and Use Committee of Institute of Medical Biology, Chinese Academy of Medical Sciences, and Peking Union Medical College, China (NO. ky201110).

### Statistical Analysis

The statistical data was analyzed using software version 17.0 (ANOVA) (IBM, Chicago, USA). Animal groups were compared by the nonparametric Mann–Whitney Test. The relationship between histological scores for the duration of DENV infection and the titer of DENV RNA in liver and brain tissue was analyzed by Spearman’s rank. The bilateral significance level was α = 0.05.

## Results

### Phylogenetic Analysis of Tree Shrew STING, MDA5 and TLR3 Gene

**Figure 1:**
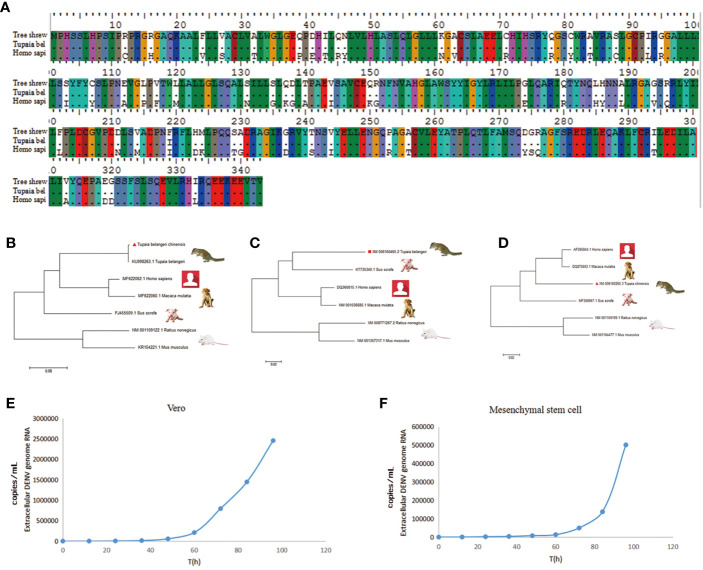
The key innate immune genes of tree shrew are genetically close to *Homo sapiens*. **(A)**, The variation of coding amino acid of STING gene in tree shrew (*Tupaia belangeri* chinensis), and *Homo sapiens*. **(B–D)**, Phylogenetic tree of STING **(B)**, TLR3 **(C)** and MDA5 **(D)** gene of *T. belangeri*, *H. sapiens*, *Macaca mulatta*, *Sus scrofa* and Mus. The phylogenetic tree was constructed using the Maximum Likelyhood phylogeny test. Bootstrap values were set for 1,000 repetitions. **(E, F)** Viral replication of DENV-3 in Vero **(E)** and BM-MSCs **(F)** cells. DENV-3 genomic RNA was tested from 0 to 96 h post-inoculation in culture supernatants of Vero cells and tupaia BM-MSCs.

Tree shrews are much genetically closer to primates than rodents. We validated this with several important immune genes including STING, MAD5 and TLR3 that play important role in the innate immunity against DENV. STING has dual functions in host defense, it can regulate protein synthesis to prevent RNA virus infection; on the other hand, it can regulate IFN expression to restrict DNA viruses (18). The nonstructural protein of DENV, NS2B-NS3 protease complex cleaves STING in permissive human primary cells; while, murine STING is resistant to the NS2B3 cleavage (19, 20). The length of STING gene is approximately 1,050 bp in tree shrew, primate and rodent species. Melanoma differentiation-associated gene 5 (MDA5) is a viral RNA receptor and triggers robust innate immune responses to DENV. Phylogenetically, STING, TLR3 and MDA5 genes of tree shrew is more closely related to *H. sapien* and *M. mulatta* than *S. scrofa* in the ML tree (maximum likelihood tree). Indeed, STING and MDA5 genes of tree shrew had a much high similarity to humans than rat. The homology of STING and MDA5 gene between human and tree shrew was as high as 85%, while that is only 75–76% between human and rat (**Figures 1A–D**, **Tables S1–3**).

### DENV-3 Infection of Primary Tupaia Bone Marrow Mesenchymal Stem Cells

The proliferative of DENV-3 viruses in tupaia BM-MSCs (Bone marrow mesenchyml stem cell) was investigated every 12 h. As shown in **Figures 1E, F**, DENV virus began to productively proliferate in tupaia BM-MSCs at 4 days after infection. Vero cells were used as positive controls (**Figures 1E, F**). Using the reverse-transcription quantitative (RT-qPCR) methods, the presence of DENV-3 genomic RNA were tested from 0 to 96 h post-inoculation in the culture supernatants of tupaia BM-MSCs and Vero cells. As shown in **Figures 1E, F**, the DENV-3 RNA was positive in all the culture supernatants of tupaia BM-MSCs and Vero cells beginning from 60 h post-infection and steadily increase thereafter (**Figures 1E, F**).

### Fever and Death Induced by DENV

**Figure 2:**
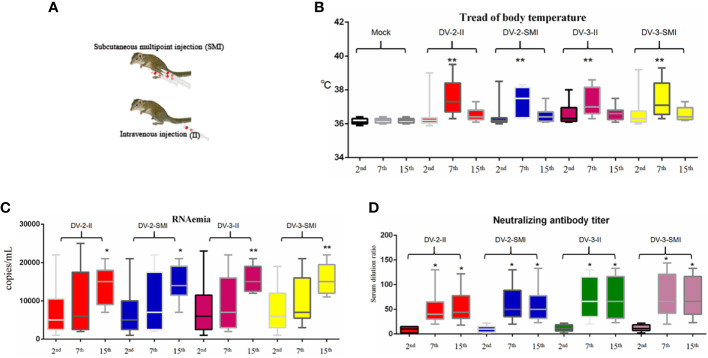
Fever, viremia and neutralizing antibody level of DENV-2 and DENV-3-inoculated tree shrew (n = 9 per group). **(A)** Schematic diagram of subcutaneous multipoint injection (SMI) and intravenous injection (II) in tree shrew. **(B)** The fever situations of the entire experimental process. The results from each group were compared using Student’s t test. **P <0.01. **(C)** The viral load in the serum. The viral RNA copies wERE tested at the day after blood collection on the 2nd, 7th and 15th days post injection. **(D)** The neutralizing titer in the serum after injection was observed on the 2nd, 7th and 15th days. The results from each group were compared using Student’s t test. *P <0.05, **P <0.01, ***P <0.001.

In this study, thirty-six tree shrews were injected with 150 µl (1 × 10^4^ PFU/ml) of DENV through intravenous injection (II) and subcutaneous multipoint injection (SMI) (performed on the abdomen of the tree shrew that three points close to each other) (**Figure 2A**). The normal temperature of the tree shrew is 36.5 ± 0.5°C. The temperature was measured among different time periods early (2nd d), middle (7th d) and later (15th d) after injection, and it was generated through infrared thermometer for each animal. The results indicate that there were no significant differences among the DV-2-II, DV-2-SMI, DV-3-II, and DV-3-SMI groups. The DV-2-II group had a maximum mean temperature; DV-2-SMI was the minimum temperature group. The body temperature of four tree shrews was over 39 °C (DV2-II, DV2-SMI, DV3-II, DV3-SMI) from 2nd to 8th days and that of eight tree shrews was over 38 °C (DV2-II (3), DV2-SMI (1), DV3-II (2), DV3-SMI (2)) after the virus injection from 3rd to 10th days. One tree shrew presented with dengue fever on the next day after DENV-2 injection, while up to six tree shrews presented with dengue fever on the 28th day. Statistically significant differences in the body temperature between the infected and non-infected animals were observed at different time points (**P <0.01) (**Figure 2B**). A total of five deaths occurred within 48 days after the injection (DV2-II (1), DV3-II (2), DV3-SMI (2)), three males and two females died on days 3, 7, 21, 34 and 37 (**Table 2**).

**Table 2:** The death after the first injection.

Time (day)	3	7	21	34	37
TS17	Death				
TS31			Death		
TS36					Death
TS40		Death			
TS44				Death	

### DENV Viremia

The dengue viral RNA in the plasma of infected tree shrews was quantified using real-time RT-PCR. Half of the tree shrews had shown viremia at the 7th and 15th days after injection (**Figure 2C**). The dengue viral RNA was not detected in the control group. The viremia of DV3-II and DV3-SMI groups were slightly higher than DV2-II and DV2-SMI groups. Using statistical analysis software, significant differences in viremia of tree shrew serum were observed at different time points among DV2-II and DV2-SMI groups (**P <0.01) (**Figure 2C**).

### Neutralizing Antibodies

Production of neutralization antibodies is a major indicator of host resistance to pathogens. Most tree shrews presented high neutralizing antibody titers at 7 and 15 days after injection. The neutralizing titers in the DV-3-II and DV-3-SMI groups were slightly higher than those in the DV-2-II and DV-2-SMI groups. There was no difference between the DV-3-II and DV-3-SMI groups, and the lowest neutralizing titer was observed in the DV-2-II group. A much higher neutralizing antibody titer was observed on the 7th and 15th days than that on the 2nd day. Using statistical analysis software, significant differences in the neutralizing titer of tree shrews were observed at different times among DV2-II and DV2-SMI groups (*P <0.05) (**Figure 2D**). The neutralizing titer was not detected in the control group and the groups of DV3-II and DV3-SMI were slightly higher than DV2-II and DV2-SMI.

### Blood Chemistry

**Figure 3:**
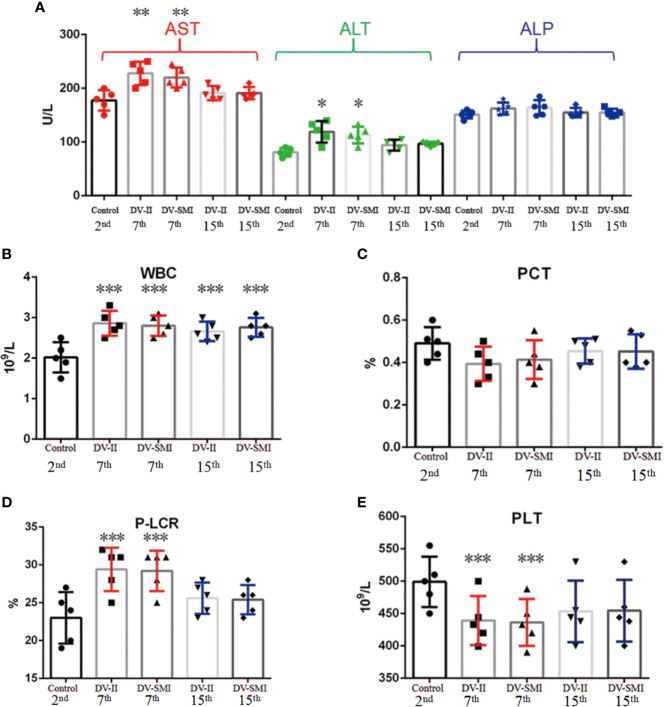
Hematological analysis and concentration of liver enzymes in the blood of DENV-2 and DENV-3-inoculated tree shrew (n = 9 per group). **(A)** The biochemical indicators AST, ALT and ALP on the different time periods early (2nd d), middle (7th d) and later (15th d) after injection. **(B–E)** The blood tests for WBC, PCT, P-LCR and PLT on the different time periods early (2nd d), middle (7th d) and later (15th d) after injection. *P <0.05, **P <0.01.

The WBC (white blood cell), RBC (red blood cell), HGB (hemoglobin), HCT (hematocrit), PLT (Platelets), MPV (mean platelet volume), PCT (thrombocytocrit), MCV (mean corpuscular volume), MCH (mean corpuscular hemoglobin), MCHC (mean corpuscular hemoglobin concentration), RDW-SD (red blood cell distribution width), RDW-CV (red blood cell-CV), PDW (platelet distribution width) and P-LCR (platelet large cell ratio) were measured in fourteen tree shrews. No significant changes were observed in RBC, HGB, HCT, MPV, MCV, MCH, MCHC, RDW-SD, RDW-CV and PDW, but changes were observed in WBC, PLT, PCT and P-LCR among different time periods early (2nd d), middle (7th d) and later (15th d). The WBC and P-LCR increased during the middle periods of infection and fell thereafter when compared to uninfected controls; however, the PCT and PLT showed opposite trends (**Figures 3B–E**). Clinically, approximately one-third of dengue fever patients show mildly or moderately elevated alanine aminotransferase (ALT) levels. At three blood collection times including early (2nd d), middle (7th d) and later (15th d) phases after DENV injection, the biochemical indicators AST, ALT and ALP were detected in the sera of tree shrews. AST and ALT levels were elevated in the second and third weeks of infection when compared to uninfected controls and wend down to basal levels thereafter (**Figure 3A**).

### DENV Dissemination to Liver and Brain

Routine examination of the cerebrospinal fluid is usually normal, and intracranial hemorrhage or brain edema in DENV patients is rare. From cerebrospinal fluid, the dengue virus can be isolated, and the viral nucleic acid or specific virus antigen can be detected. The liver histopathology of dengue fever cases shows nonspecific lesions. The cytoplasm of most of the liver cells shows a balloon-like and slight eosinophilic change, accompanied by an individual eosinophilic body.

**Figure 4:**
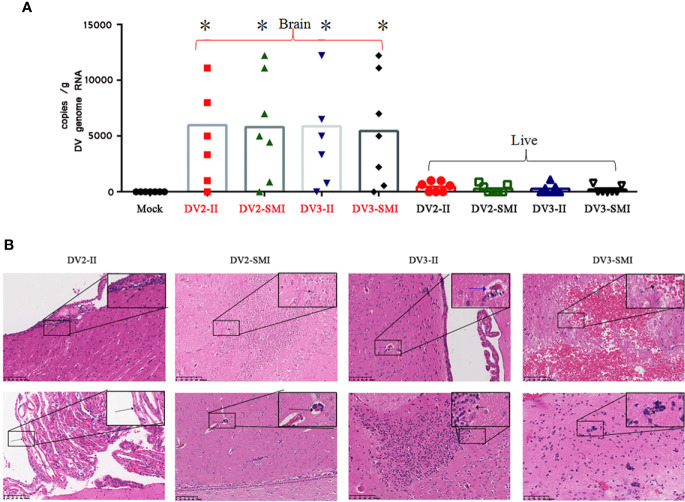
Viral RNA copies and H&E analysis in tissues of DENV-2 and DENV-3 infected tree shrew. **(A)** Genome RNA of DENV in the liver and brain. Circle dots represent the control group. 48 days post infection. High viral RNA copies were detected in the brain. The results from each group were compared using Student’s t test. *P <0.05. **(B)** Histopathology aspects of *Tupaia chinensis* brain tissue 48 days post infection of DENV-2 (DV2) and DENV-3 (DV3). DV2-II, a small number of inflammatory cells were seen locally near the ventricle in brain tissue (Black arrow) and local proliferation of arachnoid endothelial cells in brain tissue (Black arrow) were observed. The Blood vessel sleeve (Black arrow) and glial nodule (Blue arrow) were observed in brain tissue of DV2-SMI. DV3-II, glial nodules (Black arrow), individual vascular sleeves (Blue arrow) and proliferation of keratinocytes (Black arrow) in brain tissue were observed. DV3-SMI, hemorrhagic foci of brain tissue (Black arrow) and multiple glial nodules in brain tissue (Black arrow) were observed. *P <0.05.

The DENV RNA of tree shrew liver and brain were examined at the 48th day after injection. Viral RNA was detected in the brain of tree shrews infected with either DENV-2 or DENV-3. However, DENV-2 and DENV-3 RNA were barely detected in the liver. Using statistical analysis software, significant differences in the viral load were observed in the liver and brain of tree shrews among DV2-II and DV2-SMI groups (*P <0.05) (**Figure 4A**).

### Histopathology in the Brain of Tree Shrews After Infection With DENV-3 and DENV-2

The bleeding lesions in the intravenous infection group were more severe than those in the subcutaneous infection group in brain tissues. Local subarachnoid hemorrhage, local proliferation of glial cells in the cortex and parenchymal hemorrhage of brain tissue were found in the brain tissues of tree shrews infected by DENV-3 (**Figure 4B**). Individual glial nodules and individual vascular sleeves were observed in local ventricle. Severe bleeding was observed in three tree shrews of intravenous infection group, and only one showed severe bleeding in the subcutaneous infection group. Moreover, one tree shrew showed edema of choroid plexus and ependymal epithelial cells in the intravenous infection group.

## Discussion

There have been some studies on the DENV infection model in NHPs. Development of a suitable animal model for DENV disease is important for understanding pathogenesis (7). These studies included those related to the effect of experimental and natural infections, studies of the immune response of animals infected with DENV, and the evaluation of several candidate dengue viral vaccine formulations (21–25). The kinetics of dengue viremia and the generation of dengue virus specific antibody in these monkeys have been shown to generally resemble to that observed in human dengue virus infection (22). However, even many recent efforts have been made to improve the NHP models, no overt clinical symptoms develop, could sustain viral replication but at a low level, and do not develop vascular leak or syndromes resembling DHF or DSS (6, 26, 27).

As for mouse models of DENV infection, neurological disease and antibody dependent enhancement (ADE) could be developed in AG129 mice, which lack IFN-α/β and -γ receptors (28, 29). The AG129 mice infected with mouse-adapted DENV strains could also lead to a lethal vascular leak syndrome (30–32). SCID mice have also been used to assess vaccine effectiveness after DENV challenge (33–35). Symptoms of human related clinical diseases have not been observed in immunocompetent mouse models, but neurotropic disease could be developed (36, 37). A research group also tried to develop new mouse models by knocking in human STING, which is susceptible to cleavage by the DENV NS2B-NS3 protease, and more susceptible to DENV infection (19, 20, 38, 39).

Several virus infection models have been established in tree shrews, such as HEV, HAV, HBV, HSV, HCV and HDV (40). It is also reported that infection model of Zika virus had been established in tree shrew (12, 13). Here, we reported an infection tree shrew animal model of DENV. In this study, tree shrews experienced fever, death, viremia and abnormal aminotransferase levels after infection with DENV, and showed resemble clinical manifestations as in humans.

Notably, viremia was detected in tree shrews at a lower level, which may account for the relatively benign overall disease course in these animals, whereas the lower viral load in tree shrews is sufficient to induce mild hemorrhage. Lower viremia could lead to their prolonging the infectious reservoir (41). The presence of antibodies was almost non-detectable on the second day, and the purpose of antibody detection on the second day was to conduct a control experiment. The antibody titers range from 160 to 320 in mice animal model, contrastive, the antibody titers range from 20 to 150 in tree shrew animal model (42). Neutralizing antibodies and viremia were detected in the same time frame for the reason of that haven’t produced enough high levels of neutralizing antibodies, in addition, there was a good multiplication in tree shrews of DENV infection at early stage (**Figures 2C, D**). A similar phenomenon happens to human, DENV viremia can be seen in both asymptomatic and symptomatic patients and the viral decay rate was slower in asymptomatic patients (41). Thus, this tree shrew model of DENV infection may not only provide a valuable tool for further studies on the various pathophysiologic effects of dengue virus infection, but also provide a comprehensive analysis of host–virus interactions. In addition, this model is also an important tool for the testing and evaluation of potential dengue virus vaccines and therapeutic drugs. Since antibody dependent enhancement (ADE) of second infection of DENV could be develop in AG129 mice, so it is also a promising work to develop ADE model in tree shrew in the future.

Vascular leakage is an important aspect of dengue model system (43). Severe vascular pathology of extensive microvascular permeability and plasma leakage into tissues and organs were observed in DENV infection of patients (44). It is very important and necessary to examine blood vascular leakage in tree shrews. The vascular leakage of tree shrew in DENV infection was pending experimental validation.

Central nervous system (CNS) impairment was reported in DENV infection of mice and human (36). The bleeding lesions, local subarachnoid hemorrhage, local proliferation of glial cells in the cortex and parenchymal hemorrhage of brain tissue were found in the brain tissues of tree shrews infected by DENV-3 (**Figure 4B**). In addition, viral RNA were detected in the brain, but not detected in the liver. The breaching mechanism and hyperpermeability of blood–brain barrier by DENV were primarily due to direct consequences of viral infection of endothelial cells (45).

The tree shrews used in this study were collected from the wild which possess complex genetic background. The complexity and diversity of results may be caused by the diversity of tree shrews. Gratifying, there were fever, viremia, DV proliferation and pathological changes in brain and other clinical symptoms among many tree shrews. The technology of artificial propagation of tree shrews has been realized, so in the future, when the genetic background is simpler and clear, the disease symptoms of tree shrews DENV infection model will be more uniform.

Dengue fever and neutralizing antibodies are consistently observed in the tree shrew model of dengue virus infection. These findings provide a scientific basis to establish a future mature dengue animal model as a scientific platform for the in-depth understanding of the disease pathogenesis and the development of effective therapeutic drugs and vaccines against dengue fever.

## Data Availability Statement

The raw data supporting the conclusions of this article will be made available by the authors, without undue reservation.

## Ethics Statement

The animal study was reviewed and approved by Institute of Medical Biology, Chinese Academy of Medical Sciences.

## Author Contributions

LJ: Performed the experiments, acquisition and analysis of data, drafting of the manuscript, critical revision of the manuscript. CL and QS: Technical or material support, study concept and design, critical revision of the manuscript, obtained funding, study supervision. All authors contributed to the article and approved the submitted version.

## Funding

This research was supported by the Foundation of the CAMS Initiative for Innovative Medicine (CAMS-I2M) (grant no. 2017-I2M-2-006), the National Natural Science Foundation of China (31970868), Major Projects and Key Research and Development Plans of Yunnan Province (2019ZF004), the Natural Science Foundation of Yunnan Province (2016FA029), Yunnan Health Training Project of High Level Talents (D-201605).

## Conflict of Interest

The authors declare that the research was conducted in the absence of any commercial or financial relationships that could be construed as a potential conflict of interest.

## Abbreviations

DENV, dengue virus; NHP: nonhuman primates; DF, dengue fever.
