# Supplementary material for: Tree Shrew as a New Animal Model for the Study of Dengue Virus
Source: Front Immunol. 2021 Mar 25;12:621164. doi: 10.3389/fimmu.2021.621164 (PMC8026886; doi:10.3389/fimmu.2021.621164)
Supplement: Supplementary file 1 [file DataSheet_1.doc]

Table S1. Nucleotide (NT) identity of Tree shrew, Homo and Rattus species STING gene.

|  | KU998263.1  T.b | MF622062.1  H.s | NM_001109122.1  R.n | KR154221.1  M.m | MF622060.1  Macaca. m | FJ455509.1 Sus. s |
| --- | --- | --- | --- | --- | --- | --- |
| Tree shrew^＊^ | 99 | 85 | 76 | 75 | 83 | 83 |
| KU998263.1  T.b |  | 84 | 76 | 77 | 83 | 84 |
| MF622062.1  H.s |  |  | 77 | 77 | 95 | 84 |
| NM_001109122.1 R.n |  |  |  | 88 | 75 | 77 |
| KR154221.1  M.m |  |  |  |  | 75 | 77 |
| MF622060.1 |  |  |  |  |  | 82 |

Table S2. Nucleotide (NT) identity of Tree shrew, Homo and Rattus species TLR3 gene.

|  | DQ360815.1  H.s | NM_001036685.1 Macaca. m | XM_008771267.2 R.n | NM_001357317.1 M.m | KT735340.1 Sus. s |
| --- | --- | --- | --- | --- | --- |
| XM_006160465.2 T.b | 68 | 84 | 70 | 70 | 84 |
| DQ360815.1  H.s |  | 90 | 64 | 65 | 75 |
| NM_001036685.1 Macaca. m |  |  | 80 | 80 | 85 |
| XM_008771267.2 R.n |  |  |  | 86 | 78 |
| NM_001357317.1 M.m |  |  |  |  | 78 |

Table S3. Nucleotide (NT) identity of Tree shrew, Homo and Rattus species MDA5 gene.

|  | AF095844.1  H.s | DQ875603.1 Macaca. m | NM_001109199.1 R.n | NM_001164477.1 M.m | MF358967.1 Sus. s |
| --- | --- | --- | --- | --- | --- |
| XM_006160265.3 T.b | 86 | 88 | 69 | 66 | 85 |
| AF095844.1  H.s |  | 98 | 77 | 74 | 87 |
| DQ875603.1 Macaca. m |  |  | 81 | 79 | 87 |
| NM_001109199.1 R.n |  |  |  | 87 | 80 |
| NM_001164477.1 M.m |  |  |  |  | 77 |

Notes: ^＊^, The sequencing of Sting gene of tree shrews used in this study.

T.b, Tupaia belangeri

H.s, homo species

R.n, Rattus norvegicus

M.m, Mus musculus

Macaca. M, Macaca mulatta

Sus. s, Sus scrofa
